# Announcement: 6^th^ International Symposium on Metal Ions in Biology and Medicine

**DOI:** 10.1155/MBD.1999.371

**Published:** 1999


### 6TH INTERNATIONAL SYMPOSIUM ON METAL IONS IN BIOLOGY AND MEDICINE
### 7 - 10 May 2000
### Caribe Hilton, San Juan, Puerto Rico, USA

The *6th International Symposium on Metal Ions in Biology and Medicine* (6th ISMIBM) will be held on the "Island of Enchantment,"Puerto Rico, and its beautiful city of San Juan. This marks the beginning of a new era for our Symposium series, as we celebrate the 10th anniversary since the first Symposium in Reims, France. The 6th ISMIBM will be our first International Symposium in the 21st Century. Therefore, it has particular significance for the scientists coming from all corners of the world.
This Symposium will celebrate advances made in understanding the roles of metal ions and trace elements in biology and medicine. The Symposium organizers have assembled an international group of speakers to conduct short courses, plenary sessions, concurrent sessions and poster presentations. Attention will be directed to all metal ions and trace elements with special focus on the health effects of arsenic. We invite you to join us to hear about these new advances in metal ions research as they apply to analysis, environmental assessment, toxicology, biology, and health. To round out the program we want you to share the unique insight gained from your innovative research.

**HISTORICAL PERSPECTIVE:**
The *International Symposium on Metal Ions in Biology and Medicine* originated in 1990 in Reims, France, and has been held every two subsequent years. The chronological sequence of previous conferences is listed below:

| *Location & Year* | *Chairpersons* |
|---|---|
| 1st: Reims, France, 1990 | Dr. Ph. Collery and Dr. J.C. Etienne |
| 2nd: Loutraki, Greece, 1992 | Dr. J. Anastassopoulou and Dr. T. Théophanides |
| 3rd : Montreal, Canada, 1994 | Dr. N.A. Littlefield and Dr. L. Poirier |
| 4th : Barcelona, Spain, 1996 | Dr. J.L. Domingo, Dr. J.M. Llobet, and Dr. J. Corbella |
| 5th : Munich, Germany,1998 | Dr. P. Brätter, Dr. V. Negretti de Brätter, and Dr. P. Schramel |

Members of the International Scientific Committee are named by the new organizers. Former chairpersons remain permanently in the International Scientific Committee.

**OBJECTIVES OF THE SYMPOSIUM**:
- To promote the exchange and dissemination of scientific knowledge about trace elements and metal ions and their role in biological processes and the etiology of disease, including cardiovascular disease, diabetes, and cancer.
- To explore new advances, applications and uses of metal ions in medical research, including clinical applications, aging, nutrition, and cancer prevention.
- To discuss new technological developments for the analysis and speciation of trace elements and metal ions.
- To foster discussion on environmental and public health research needs.

**AUDIENCE:**
The 6th ISMIBM will be of interest to a wide scientific audience including:
- Medical scientists, health professionals, toxicologists, pharmacologists, nutritionists, and others interested in the effects of metal ions, trace elements, and minerals on human and environmental health.
- Environmental and industrial scientists, epidemiologists, geologists.
- Biochemists, biologists, chemists.
- Microbiologists, geneticists, molecular biologists, cell biologists.

**COMMITTEES:**
*Chairman, International Scientific Committee*:
Dr. José A. Centeno

Dept. of Environmental and Toxicologic Pathology
Armed Forces Institute of Pathology (AFIP), Washington, DC 20306-6000, USA
Phone: (202) 782-2839; Fax: (202) 782-9215; Email: centeno@afip.osd.mil

*Co-Chairman*: Dr. Philippe Collery (France)
Institut International de Recherche sur les Ions Métalliques
Email: Philippe.collery@wanadoo.fr

*Honorary Chairpersons*:
Dr. Florabel G. Mullick (USA)
Center for Advanced Pathology, AFIP
Prof. Dr. Jean-Claude Etienne (France)
Institut International de Recherche sur les Ions Métalliques and
Conseil Régional de Champagne Ardenne

*General Secretary*:
Dr. Lylia Khassanova (Russia)

*International Scientific Committee:*

C. Abernathy (USA)

C. Alpoim (Portugal)

J. Anastassopoulou (Greece)

O. Andersen (Denmark)

V.H. Aposhian, (USA)

J.R. Arthur (Scotland, UK)

G. Beckett (Scotland, UK)

A. Berthelot (France)

P. Brätter (Germany)

S. Caroli (Italy)

M. Cebrian (Mexico)

G.F.Combs (USA)

B. Conard (Canada)

J. Corbella (Spain)

R. Cornelis (Belgium)

M. Costa (USA)

R. Deloncle (France)

B. Desoize (France)

F.A. de Wolff (The Netherlands)

J.L. Domingo (Spain)

J. Durlach (France)

B. Farzami (Iran)

M. Fields (USA)

M.F. Flores-Arce (Mexico)

B. Fowler (USA)

M. Gielen (Belgium)

O. Guillard (France)

K. Irgolic (Austria)

V. Kalfakakou (Greece)

K.S. Kasprzak (USA)

N.A. Littlefield (USA)

J.M. Llobet (Spain)

D.N.G. Mazumder (India)

B. Michalke (Germany)

V. Negretti de Brätter (Germany)

J. Neve (Belgium)

G. Nowak (Poland)

A.R. Oller (USA)

A. Pineau (France)

L.A. Poirier (USA)

P. Schramel (Germany)

S.K. Shukla (Italy)

M. Simonoff (France)

F.W. Sunderman, Jr. (USA)

K.T. Suzuki (Japan)

T. Tchernitchin (Chile)

Th. Théophanides (Greece)

D. Templeton (Canada)

M. Vahter (Sweden)

G. Vernet (France)

M.P. Waalkes (USA)

G. Zaray (Hungary)

B.S. Zheng (China)

*Organizing Steering Committee:*
Dr. Herman Gibb (US EPA), Co-Chair
Dr. Michalann Harthill (USGS), Co-Chair
Dr. Robert B. Finkelman (USGS)
Dr. David Longfellow (NIH-NCI)
Dr. David Reese (US EPA)
Dr. Kenneth Cantor (NIH-NCI)
Dr. Claudia Thompson (NIH-NIEHS)

*Local Organizing Committee* (Puerto Rico):

Dr. Jaime Matta, Co-Chairman, Ponce School of Medicine

Dr. Jorge I. Vélez-Arocho, University of Puerto Rico – Mayagüez

Prof. Sylvia Marquéz de Pirrazi, University of Puerto Rico-Mayagüez

Dr. Samuel P. Hernández, University of Puerto Rico - Mayagüez

Dr. Braulio Jiménez, School of Medicine of Puerto Rico

Dr. Miguel Sastre, University of Puerto Rico – Humacao

Prof. José Cintrón, Ana G. Méndez University System, CUE

Dr. Ismael Scott, University of Puerto Rico-Mayagüez

**JOINTLY SPONSORED BY:**

Armed Forces Institute of Pathology (AFIP)
American Registry of Pathology (ARP)
Institut International de Recherche sur les Ions Métalliques (IIRIM-Reims,France)
University of Puerto Rico at Mayagüez (UPRM)
Ana G. Méndez University System of Puerto Rico (AGMUS)
Jackson State University – School of Science & Technology (JSU)
U.S Environmental Protection Agency (US EPA)
U.S Geological Survey (USGS)
National Cancer Institute (NCI)
National Institute of Environmental Health Sciences (NIEHS)
American Association for the Advancement of Science (AAAS)-Caribbean Division

**UNDER THE AUSPICES OF:**

Federation of European Societies on Trace Elements and Minerals (FESTEM)
Nickel Producers Environmental Research Association (NiPERA)
National Natural Science Foundation of China (NNSFC)
Center for Hemispherical Cooperation on Science and Engineering (UPRM)
Conseil Régional de Champagne Ardenne (CRCA-Reims, France)
Ponce School of Medicine, Puerto Rico (PSM-PR)
Societé pour le Developpement des Recherches sur le Magnesium (SPDRM)
International Association of Geochemistry and Cosmochemistry (IAGC)
Society of Environmental Toxicology and Chemistry (SETAC)

**SYMPOSIUM SERVICE OFFICE:**

American Registry of Pathology
Department of Medical Education
Armed Forces Institute of Pathology
Washington, DC 20306-6000
Toll Free Tel: (800) 577-3749 (U.S. only); Tel: (202) 782-2637
Fax: (202) 782-5020; International Toll Free Fax: (877) 891-3482

**SCIENTIFIC PROGRAM**

The Scientific Program will be composed of General Plenary Sessions (3), concurrent sessions, and poster presentations. Oral (platform) presentations will be 15 minutes in length with five minutes for discussion. Posters will be exhibited for the first two days.

***Scope of the Scientific Program:***

The following topics are examples of the intended breadth of the Symposium:

Metals and Environmental Health
Speciation of Metals and other Elements
Metals in Clinical Applications
Molecular Toxicology of Metals
Carcinogenesis of Metals
Epidemiology and Occupational Health
Metals and Disease: Environmental and Toxicologic Pathology
Health Effects of Arsenic
Metals and Aging
Metals and Homeostasis
Effects of Low and High Nutritional Trace Elements Intake
Risk Assessment of Trace Element Status and Health
Toxicity of Metals
Metals and Hormone Receptors
Metals and Chelation Therapy
Metals and Enzyme Activity
Advanced Methods for the Analysis of Trace Elements and Metal Ions

***Preliminary Program:***

Sunday, May 7th:

**Registration and Special Program with Short Courses**

Welcome Reception ("Conquistadores" Night, San Geronimo Fort)
(hosted by the Ana G. Méndez University System of Puerto Rico)

Monday, May 8th:

**Registration**

**General Plenary Session: Invited Speaker from the U.S. National Institute of Environmental Health Sciences (NIEHS)**

An overview of activities at the NIEHS on human health and environmental effects of metal ions

**Concurrent Sessions (4) and Poster Session I**

**Get Together Party: Puerto Rican Cultural Night**

Tuesday, May 9th:

**Registration**

**General Plenary Session: Invited Speaker from the U.S. Environmental Protection Agency (US EPA)**

An overview of activities at the US EPA on human health and environmental effects of metal ions

**Concurrent Sessions (4) and Poster Session II**

**Symposium Gala Dinner** (hosted by the University of Puerto Rico at Mayagüez)

Poster Awards (hosted by FESTEM)

Wednesday, May 10th:

**Registration**

**General Plenary Session: Invited Speaker from the U.S. Department of the Interior**

An overview of activities at the US Geological Survey on fish, wildlife and human health and environmental effects of metal ions

**Concurrent Sessions (4)**

***Special Program on Short Courses:***

Short courses will be held on Sunday, May 7. The cost is $50.00 per course and includes attendance at the special session, book of lectures, and coffee breaks. Currently, the following short courses are being provided. Additional courses, if developed, can be seen at the website: www.afip.org/edu.

1. Title: ***Analytical Methods for Trace Elements in Biological Tissues and Fluids***
   Lecturers:
   Patrick J. Parsons, New York State Department of Health, Albany, New York
   Robert J. Jones, Center for Disease Control and Prevention, Atlanta, GA, USA
   Course Description: This course will describe applications of atomic spectrometry methods for determination of selected toxic elements in biological matrices.
   Time of Course: 8:30 – 11:30AM
2. Title: ***Metal Ions in Environmental Health and Disease***
   Lecturers: Robert B. Finkelman, US Geological Survey, Reston, VA, USA
   Kurt Nordstrom, US Geological Survey, Boulder, CO, USA
   José A. Centeno, Armed Forces Institute of Pathology, Washington, DC
   Michalann Harthill, US Geological Survey, Reston, VA, USA
   Course Description: This course is designed to introduce participants to geochemical/biochemical cycles of potentially toxic metals such as arsenic. We will follow the metal from its natural and anthropogenic sources to its solubilization, mobilization, and biological uptake under different environmental conditions, and discuss potential toxicities or beneficial effects in wildlife and human health.
   Time of Course: 8:30 – 11:30AM
3. Title: ***Toxicology, Health and Remediation of Selected Contaminants in Environmental Toxicology***
   Organizers : Jackson State University – School of Science & Technology, MS
   Ana G. Méndez University System, PR, USA
   Time of Course: 8:30 – 11:30AM
4. Title: ***Medical Implications of Metal Exposures***

Lecturer: Michael R. Harburt, MD, Wayne State University, Detroit, MI
Course Description: This course will familiarize participants with the pathophysiologic effects of selected metal exposures, their medical implications, and approaches to the treatment of disease which may be caused. The lack of certainty of treatment methods based on blood and/or urine levels versus frank clinical disease will be discussed.
Time of Course: 1:30 – 4:30PM

5. Title: ***Environmental Pathology of Metal Exposures***
Organizers: Armed Forces Institute of Pathology
American Registry of Pathology
Course Description: The pathology of metal ion toxicity will be discussed. Particular emphasis will be placed on metal-induced diseases of the liver, lung, kidney, cardiovascular, and brain.
Time of Course: 1:30 – 4:30PM

**INSTRUCTIONS:**

- The organizers of the 6th ISMIBM invite all interested individuals to submit abstracts for oral or poster presentation(s). Acceptance will be based upon high quality submissions with strict relevance to this Symposium. All abstracts must be accompanied by a completed **Symposium Registration Form.**
- The official language of the Symposium is **English**.
- **Deadline for receipt of abstracts is October 15, 1999**
- Send (or email) abstract correspondence to: Dr. Jose A. Centeno, Chairman, 6th International Symposium on Metal Ions in Biology and Medicine, Armed Forces Institute of Pathology, 14th and Alaska Ave, N.W., Washington, D.C. 20306-6000.
- Abstracts may be submitted electronically to the following e-mail address: centeno@afip.osd.mil.

**SUMMARY OF IMPORTANT DATES:**

| | |
|---|---|
| Submission of Abstracts for oral or poster presentations: | October 15, 1999 |
| Notification of acceptance of abstracts: | November 15, 1999 |
| Early registration (required for ALL accepted abstracts): | January 14, 2000 |
| Submission of full manuscripts: | March 3, 2000 |
| Hotel accommodations: | April 10, 2000 |

